# Older Adult Segmentation According to Residentially-Based Lifestyles and Analysis of Their Needs for Smart Home Functions

**DOI:** 10.3390/ijerph17228492

**Published:** 2020-11-16

**Authors:** Jiyeon Yu, Angelica de Antonio, Elena Villalba-Mora

**Affiliations:** 1 Madrid HCI Lab, Research Group on Human-Computer Interaction and Advanced Interactive Systems, Universidad Politécnica de Madrid (UPM), Boadilla del Monte, 28660 Madrid, Spain; angelica@fi.upm.es; 2 Centre for Biomedical Technology (CTB), Universidad Politécnica de Madrid (UPM), Pozuelo de Alarcón, 28223 Madrid, Spain; elena.villalba@upm.es; 3 Centro de Investigación Biomédica en Red en Bioingeniería, Biomateriales y Nanomedicina (CIBER-BBN), 28029 Madrid, Spain

**Keywords:** older people, lifestyles, residentially based lifestyles, ageing in place, quality of life, older user needs, smart homes

## Abstract

Globally, the percentage of older people in the general population is growing. Smart homes have the potential to help older adults to live independently and healthy, improving their quality of life, and relieving the pressure on the healthcare and social care systems. For that, we need to understand how older adults live and their needs. Thus, this study aims to analyze the residentially-based lifestyles (RBL) of older adults and segment them to compare and analyze the real needs of smart home functions for each group. To identify a person’s RBL, a questionnaire was designed to include questions about activities at home, social events, quality of life, etc. This study surveyed 271 older Koreans. As a result of the survey on RBL, five groups with different characteristics were clustered. Finally, each groups’ features and the differences in their needs for smart home functions were compared and analyzed. The priority of needed functions for each group was found to be significantly different. In a total of 26 smart home functions, there were meaningful differences in the needs for 16 functions among the groups. This study presents the results in South Korea, according to older adults’ RBL and their smart home needs.

## 1. Introduction

The increase in the average age of the population is not only a problem being faced in the present, but is also an inevitable challenge for the future. Almost all countries are experiencing growth in the size and proportion of older adults in their society. There were 703 million people aged 65 years or above in the world in 2019. The number of the older adults is projected to double to 1.5 billion by 2050. Meaning, the proportion is expected to rise another 16 percent by 2050, making one in six people in the world 65 years or older [1]. South Korea is also in the process of becoming an aging society. In 2020, the older population represents approximately 15.6 percent of the total population in South Korea. This is about 0.8 percent higher than the previous year, and this share has been increasing continuously since 2010. Statistics Korea predicts that South Korea will be a ‘super-aged society’ in 2026, with older people occupying a proportion of 20 percent of the total population [2]. That is, South Korea is a representative country in which aging is in progress. Also, the Seoul Metropolitan Government in South Korea announced that 7500 smart IoT devices would be installed in homes of older adults living alone each year until 2022. Since 2018, Seoul has been installing residential sensors to detect motion, temperature, humidity, and lighting in the houses of older people who are unhealthy and have no family to contact. The data is being monitored in real-time, and if abnormal signs are detected, the care workers in charge immediately establish contact, visit the house and take the necessary emergency measures [3]. Moreover, according to the National Information Society Agency in South Korea, the rate of smartphone usage at the age of 60 or older is high; reaching 85% in 2019 [4]. South Korea, which is on the verge of becoming an aging society and is making efforts to apply smart home technology to the lives of older adults, is a good context to analyze the smart home technology needs of the elderly.

The increase of the older population has a social and economic impact on our society [5]. In addition, with the COVID-19 crisis, older adults are being forced to stay home for longer amounts of time and their disconnection with external assistance services is increasing, therefore smart home technology is gaining more importance among this population.

Smart homes for older people can be a great solution in three ways: (1) Personal aspects, (2) Social aspects, and (3) Technical aspects. The perception of the elderly demographic is shifting from a passive being to an active subject. Accordingly, the concepts of active aging and active seniors have emerged. They seek to live independently and focus on the quality of their life. On the personal side, smart homes can be planned to support older adults so they can live independently and safely at home and to reduce their dependence on other healthcare services [6]. On the other hand, smart home technology can also help them to receive medical treatments safely and efficiently by permitting contact with medical facilities even while in their own home. Socially, smart homes can be an effective way to foster and improve healthy lifestyles while minimizing healthcare service costs [7]. Recently, as the damage caused by COVID-19 in nursing homes and other care facilities suggests [8], society needs to find ways to newly support this population in their own homes. In this respect, smart homes can be a technology that can have an impact not only on individuals but also at the social level of public health. In the technical terms, smart home technology is being developed and researched in regards to three major aspects: comfort, healthcare, and security services. It is becoming increasingly more common thanks to technology developments such as the Internet and sensors [9]. This technology will no longer be a particular preference for someone, but it will become a mainstream technology that anyone can apply to their home. In this context, smart home technology for the older population has enormous potential.

Nonetheless, older adults may experience difficulties while interacting with smart homes, preventing them from benefiting from the technological advances and from fulfilling their social needs. Smart homes will be more useful if the market is carefully analyzed to find and prioritize user needs. While considering these points, some smart home studies have attempted to monitor user behavior and provide services that match the pattern [6]. However, there is not enough universally applicable data for user analysis yet.

As smart homes can be a technology that complements and enhances the quality of life in healthy old age, this study suggests a way to effectively understand the need for smart home technology, according to the different RBLs of this demographic. This study will construct primary data of older users through segmentation by RBL and look at the relationship between each RBL-based cluster and their needs for smart home services.

**Figure 1:**
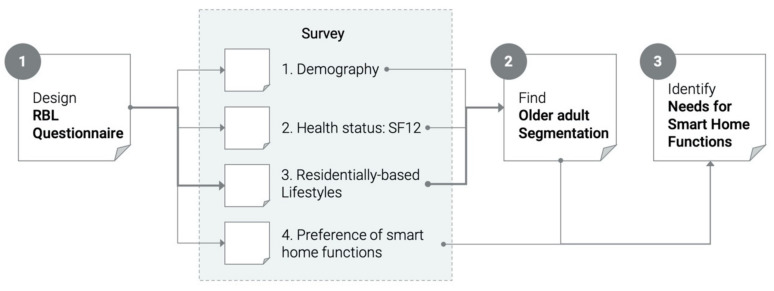
Research process and outcomes.

The purpose of this study is summarized into the following three goals (Figure 1):

(1) Design a questionnaire tool to identify RBL of older people
(2) Find older adult segmentation based on RBL for smart home planning
(3) Identify the needs of each group in regards to smart home functions and services.

This study examines the classification of lifestyles in the homes of older Koreans and their smart home function needs. It aims at building essential data and proposing a methodology applicable to other countries to help with the future design of smart homes that promotes healthy lifestyles for the older population.

### 1.1. Lifestyle of Older People

In marketing, lifestyle is often used to segment the market. In recent years, the economic capability of older adults has been strengthening, and they have increasingly become the principal subject as a consumer in the market. In this context, the concept of the grey market and the mature market was formed, and various segmentation analyses based on their lifestyles were applied to marketing [10,11].

Bone [10] found five lifestyle criteria in defining the mature segments: Income, Health, Activity level, Discretionary time, and Response to other people. Moschis et al. [12] proposed four criteria for the older consumer market: biophysical, psychological, social circumstances, and key life events. Sudbury and Simcock [13] provided a multivariate segmentation model of older consumers: (1) solitary sceptics, (2) bargain-hunting belongers, (3) self-assured sociables, (4) positive pioneers, (5) cautious comfortables. For this segmentation, they used five criteria: health and activities, social measures, psychological measures, consumer behaviors, attitudes and values, and media usage.

Additionally, some marketing researchers have suggested that the domain-specific lifestyles are the most feasible level of segmentation, providing the most meaningful results [14]. Amongst this research, there were the food-related lifestyles [15], the studies of energy-related lifestyles [16] and transport-related lifestyles [17]. For the study of smart homes, RBL and the living patterns in the house will be an important domain. Smart home functions are becoming more and more diversified, and they are being developed to control the entire house. However, until now, there has been a lack of studies on RBL for smart homes. This study proposes that in the design of an efficient smart home service for the older adults, it would be wise to identify needs according to RBL.

Although in old age physical, mental, and social weakness can be experienced, there are many individual differences, and therefore, it is important to further refine the older segment in order to find the needs of each group based on their lifestyles. Through this process, the functions and services suitable for each user’s needs can be researched beyond functionally supported smart homes.

### 1.2. Smart Homes for Older People

Recently, smart home research for the older populations has been more common. In the Sweet Home Project [18], the voice control of smart homes was tested based on several scenarios for older adults and their caregivers. As a result of this study, it was confirmed that voice control had great potential to facilitate daily life in a general sense and also for the older population. Yu et al. [6] conducted a pilot test to monitor older adults’ daily behaviors with the application of unobtrusive sensors for three months. Data such as thermal comfort, sleep, medicine compliance, water usage, doors, windows, etc. were collected, and it allowed researchers to gain a better understanding of the environments of older people. Also, there was a study completed on various healthcare technologies in smart homes for older adults [19], as well as a survey on monitoring for the detection of abnormalities by recognizing the subject’s behavior [20]. In particular, with the development of deep learning technology, recognizing the behavior of older people, detecting falls, or predicting when actions will occur have been significantly advanced in the last 3–4 years [21,22,23,24,25].

In addition to the technical aspects, there have been studies on users and usability. Meulendijk et al. [26] developed design principles for smart home services in old age for seven obtrusiveness dimensions (physical, usability, privacy, function, human interaction, self-concept, routine, sustainability) and five layers of ambient intelligence (embedding, context-awareness, personalization, adaptation, anticipation). Wong et al. [27] carried out a focus group interview to find four key intelligent attributes (autonomy, controllability of complicated dynamics, man-machine interaction, bio-inspired behavior) for smart home technology for older people. Pal et al. [28] conducted the survey to find core factors that can affect older users’ acceptance of smart home services for healthcare services.

Although various studies aimed to understand older users, there still exists a lack of understanding about this demographic. To apply the smart home technology to real life environments for them, more practical and more diverse user studies are required. For the smart homes to become a part of older adults’ lives, personalization and sufficient understanding of their residential lives are necessary. This study confirms to what extent each clustered group by RBL needs smart home services and functions. It also proves that the difference based on RBL leads to the difference in the needs for specific functions of the smart home.

## 2. Materials and Methods

### 2.1. Participants

The participants of this study are older adults in South Korea, as a representative country facing an aging society. According to the definition of the World Health Organization [29], older people are defined people aged 65 or older. Therefore, the participants were older adults aged 65 years or older living in South Korea. The survey was conducted in compliance with the sex ratio 43:57 (male: female) of the 2018 Korea Population Survey [30]. The inclusion criteria for the responses were: (1) Age of 65 or older; (2) No missing answers to any questions; (3) Response received within the survey period. The survey was first tested during one week to adjust the questions and wording to improve understanding. Responses received over the test were discarded. Afterwards, we collected a total of 271 valid answers during four consecutive weeks. A total of 116 male respondents and 155 female respondents participated.

### 2.2. Instruments

The designed questionnaire consists of four parts: (1) demography, (2) residentially-based lifestyle, (3) health status, and (4) preference for smart home functions. The demographics section consisted of eight items: age, gender, marital status, primary caregiver, home type, the average daily time spent out of home, education, and monthly household income.

The RBL questionnaire was composed through existing study analysis [10,12,13,31,32,33,34,35,36]. It was designed to check the lifestyle patterns in residential life in three aspects: (1) quality of home life indicators (general frequency 1: rarely—5: always), (2) daily home activities (daily frequency 1: never—5: more than 3 h), and (3) social events at home (monthly frequency 1: less than 1–5: more than 4 times). More details are provided in Section 2.2.1.

In addition, as older people’s health can be an important indicator, a health questionnaire was applied using the 12-item Short Form Health Survey (SF-12) in a separate section for comparison with each group. SF-12, which has proven reliability and validity in previous studies, is a tool developed to measure respondents’ health status in a short time by extracting 12 questions from SF-36. A higher score means better health and a higher quality of life. And the physical component summary (PCS) and mental component summary (MCS) are calculated through twelve questions in eight health areas, and it can be evaluated based on the U.S. average score of 50 [37].

In the preference of smart home functions section, the functions were extracted based on meta-analysis of existing smart home studies (primary articles: 42, backward citation: 101) [38]. All questionnaires were carefully revised with a gerontology research expert and a smart home technology expert.

#### 2.2.1. Residentially-Based Lifestyle Questionnaire

In this study, it was very important to develop an RBL measurement tool that could be the basis for clustering. To this end, measurement items were constructed by analyzing studies related to the lifestyles of older people [10,12,13,31,32,33,34,35,36] (Appendix A). To further define the concept of residential life, existing studies on housing and residential lifestyle were also analyzed [39,40]. By analyzing the related works, the pertinent elements were defined in order to create RBL questionnaire for older people: quality of daily life, The need for help in home life, daily activities ((a) leisure, (b) housework, (c) relaxation) at home, and social events at home.

First, the quality of daily life section includes questions about basic nutrition and sleep quality assessments. At the same time, it also looks at chronic diseases and regular use of medicines to assess the quality of life according to health conditions. Second, the respondents were asked about home activities for which they need help. The activities are grouped into four areas: housework, grooming, cooking/shopping, and care. Third, the respondents were asked about their activities at home to grasp their life patterns through the frequency of activities. Home activities are grouped into three areas: (1) leisure, (2) housework, (3) relaxation. The detailed activities were constructed referring to related studies on the activities of older users’ daily life [41,42,43,44]. Lastly, the questionnaire asked the frequency of visits by outsiders to evaluate social events occurring at home. The completed RBL questionnaire can be seen in Appendix C.

#### 2.2.2. Questionnaire on Preference of Smart Home Functions

In this study, it was important to extract and organize the smart home functions based on the most common services. Marikyan et al. [38] conducted a systematic review of existing literature studies (primary articles: 42, backward citation: 101) on smart homes in 2019. Based on this research, the main functions of smart homes were summarized. The study [38] identified five major services and 13 smart home functions. On the basis of this data, four services were considered except for the consultancy service, which is only indirectly related to users. Three functions from the support service were also excluded because they are only useful in specific situations. As a result, a total of 26 detailed functions could be determined. Also, a simple description of each item was added to help older adults understand the functions (Appendix B). The preference was measured with a 5-point Likert scale on each detailed function: 1—not necessary, 2—a little necessary, 3—necessary, 4—very necessary, 5—most necessary.

### 2.3. Procedure

This survey was conducted as a non-face-to-face tool as an online survey due to consideration for the COVID-19 pandemic situation, the smartphone penetration rate (85%, for participants over 60 years old), and Internet access rate (99.3%, over 60 years old) for the elderly in Korea [4]. The questionnaire was initially written in MS word and then was rewritten and distributed with the online survey tool called Google Forms. The questionnaire was structured to make it easy to respond to the questions using mobile phones.

In addition, when the survey was distributed, a guide to the purpose of the study and an informative note stating that individual responses would not be used for any purpose other than to collect data for the study were distributed together. Responses were collected for a set period of 4 weeks from 18 April to 18 May 2020. The responses were stored and analyzed anonymously. Also, we did not ask for any information that could identify the respondents. All respondents were older adults who were not patients and this survey did not involve any interventions or treatments.

Accordingly, our research was considered an exemption case, as specified by the National Bioethics Committee ((1) It does not include any physical interventions directly with the subject for research purposes nor interventions such as manipulation of his/her environment. (2) It does not include direct interactions during interviews, surveys, nor behavioral observations with study subjects for research purposes. (3) It does not use information (name, telephone number, id number, etc.) that can directly or indirectly identify who the subject is) (http://www.irb.or.kr/UserMenu01/Exemption.aspx), which means that we were able to conduct the research without the approval of an ethics committee.

### 2.4. Data Analysis

In RBL, the seven main factors were extracted through PCA (principal component analysis). For reliability analysis of these factors, the internal consistency was verified with Cronbach’s alpha value. Based on RBL factors, K-means clustering was able to construct segmentation by similar patterns and characteristics. After that, the demographic characteristics and health status of each group were compared. Through the questionnaire of the preference of smart home functions, each group’s needs for detailed functions were measured. To prove significant differences among groups, chi-square verification was applied to the demographic questionnaire, and for health scores and the smart home needs scale, post-validation was conducted with ANOVA analysis.

## 3. Results

### 3.1. Principal Component Analysis on Residentially-Based Lifestyles

The mean Kaiser-Meyer-Olkin (KMO) for the RBL test was 0.65, being above the 0.5 threshold in all measurement items. Therefore, it is considered suitable for factor analysis according to Williams et al. [45]. Bartlett’s sphericity test showed that all items were meaningful (1149.457, *p* = 1.281571 × ^−99^). A factor extraction model of principal component analysis (PCA) was selected and analyzed through Varimax rotation with a method of orthogonal rotation. Table 1 shows the principal component analysis results to verify the validity and reliability of RBL. Considering that this questionnaire was newly created based on some existing studies, the elements with a factor loading over 0.3 could be accepted. As a result, seven factors were determined: Quality of life, Housework, Need for help in home life, Health state-related quality, Leisure at home, Social event at home, and Relaxation at home. This shows that the classification is almost the same as the sections from which the questionnaire was composed. The composition of each factor is shown in Table 1.

After analysis, each factor’s reliability was confirmed by the Cronbach’s alpha value. Cronbach’s alpha value is about 0.6 and above, except for Relaxation at home (0.49). Each factor has an eigenvalue of 1 or more, and the total variance explained by these factors is 52%.

### 3.2. Segmentation Based on Residentially-Based Lifestyles

**Figure 2:**
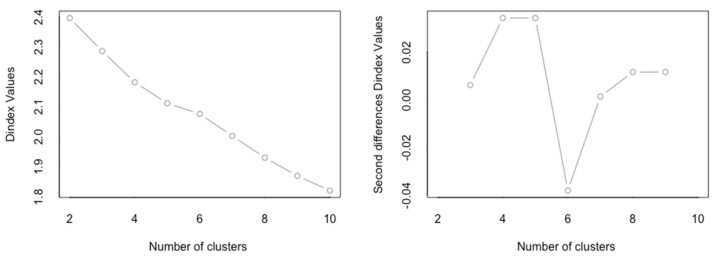
Number of clusters using NbClust.

For segmentation, non-hierarchical K-means cluster analysis was conducted based on RBL factors. Using the NbClust library of the R program (Figure 2), we identified the elbow point where the total error value was minimum. It was judged that the classification into five clusters was the most stable and convincing.

**Figure 3:**
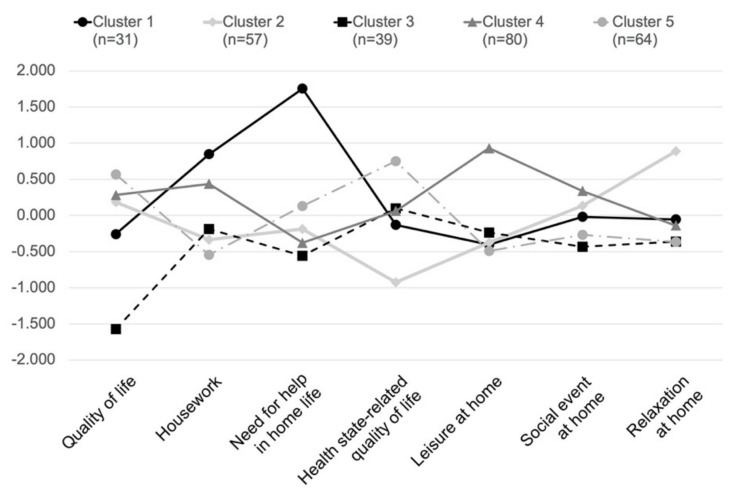
Clusters for residentially-based lifestyle of older people.

Table 2 shows the results of the clustering defined into five groups. In the case that a factor has a relatively large cluster center value, it can be characterized as a cluster that is positively affected by the factor. We conducted ANOVA analysis to explore the differences in the RBL factors, according to the clustering. The analysis shows that six factors have significant differences: 1. Quality of life (24.44 *** *p* < 0.001), 2. Housework (10.52 **, *p* < 0.01), 3. Need for help in home-life (31.88 ***, *p* < 0.001), 4. Health state-related quality (111.4 ***, *p* < 0.001), 5. Leisure at home (6.155 *, *p* < 0.05), 6. Social event at home (0.661, *p* = 0.417), and 7. Relaxation at home (54.31 ***, *p* < 0.001). The difference between the factors for each group can be seen in Figure 3.

The characteristics and lifestyle patterns of each cluster were defined and named based on the distinguishing factors. Cluster 1 “Tired of housework” is a group of people who spend a lot of time doing housework and need help with a lot of physical work. Cluster 2 “Just rest” is a group of people who are used to resting most of the time at home. They have the lowest health-related quality of life. Cluster 3 “Poor quality of life” is a group with the lowest quality of daily life, and is not very active at home. Cluster 4 “Active at home” is a group of people who spend time with various activities at home, and they have many visitors. Cluster 5 “High quality of life and good health” is the group with the highest proportion of men and the highest physical health. They have a high health-related and basic life quality.

Table 3 shows the characteristics of each cluster on the demography. For each group, there are significant differences in Gender and Income. In Clusters 1 to 4, the proportion of women is higher, whereas in Cluster 5 “High quality of life and good health”, the proportion of men (59.4%) is higher. Cluster “Tired of housework” has the highest rate of over 75 years of age, and Cluster “Poor quality of life” has the highest rate of under 70 years. Also, in Cluster “Poor quality of life”, the rate of married status is the lowest, and the rate of divorce or bereavement is relatively higher than in other clusters. In the same vein, the rate of living alone or with other family members, not a spouse, is also relatively high.

As shown in Table 4, the ANOVA analysis results show that there are significant differences in each group in the health index: physical component summary (PCS) and mental component summary (MCS). The PCS score of “Just rest” with the lowest health-related quality is the lowest, and “High quality of life and good health”, with the highest health-related quality, has the highest physical score. Moreover, “Poor quality of life”, which has the lowest basic quality of life and the least number of social events at home, also has the lowest MCS. On the other hand, “Active at home”, which spends a lot of time with various activities at home and has many visitors, is the group with the highest MCS.

### 3.3. Needs for Smart Home Functions for Each Group

Finally, the perceived needs for smart home functions of each cluster were compared. The respondents could select “1: not necessary” to “5: most necessary” for each function, and a higher number is interpreted as a higher level of need (Table 5).

The results of ANOVA analysis confirm that there are significant differences in a total of 16 smart home functions for each group. In particular, the smart home needs of “Tired of housework” are high, and “High quality of life and good health”, which is a group composed of men who do not spend much time at home, doesn’t need the functions very much.

The 16 functions with significant differences are described as follows. Reminder to take medicine function is most needed by “Tired of housework” and “Just rest”. These two groups have relatively low physical health. On the other hand, “High quality of life and good health”, which is the most physically healthy group, has minimal need for this function. Automatically checked postbox function is most needed in “Tired of housework”, and then “Just rest” and “Active at home” groups as well. For the Automatic heating function, the needs of “Tired of housework” and “Just rest” are also high. In Gas leak detection/Smoke detection function, all groups need this function, and it is the most required function by “Tired of housework”. In Remote controlled electricity function, all groups also respond that it’s important. This function takes the highest priority in “Poor quality of life” and “Active at home”. Remote controlled light is shown to be needed in three groups: Tired of housework, Just rest, and Active at home. For Light mode settings function, the need from “Active at home” is the highest. Because people from the “Active at home” group spend a lot of time at home, they are also interested in entertainment-related functions. For TV auto-play and notifications from the My channel function, “Tired of housework” and “Active at home” need it more than other groups. For the Fall detection function, most of the groups say that it’s necessary, and this function is of especially high priority for the “Tired of housework” group. Regarding the health-related features such as the Sensors you can wear as clothes and Telehealthcare, it is considered to be needed in the following order: “Tired of housework”, “Just rest”, “Active at home”, “Poor quality of life”, “High quality of life and good health”. Although the “High quality of life and good health” group is less interested in other functions, it shows a little more interest in health-related functions. In Mental health detection function, participants responded that it is a necessary function in the following order: Tired of housework, Active at home, Just rest, and Poor quality of life. Moreover, in “Poor quality of life”, the group with the lowest mental health scores, this function takes a relatively high priority. In Home Management Assistant Robot function, the need for this function from “Just rest” is higher than other groups. In the case of Equipment to help you up and down stairs, people from “Tired of housework” and “Just rest”, who have a relatively low physical score, they need it more, and it is not necessary for the rest of the groups. Regarding Robot like a friend function, the needs of all groups are low, and Call the family in case of emergency function is a necessary function in all groups. In particular, “High quality of life and good health” group selected this as the highest priority function.

Table 6 is a list of score-ordered functions for each group, among the functions that scored 2.5 or higher.

The amount of need and priority of the functions for each group are all different. In the case of “Tired of housework”, the average age is high, and the PCS (44.915) and the MCS (48.272) are low, and they need the smart home functions the most. In particular, they are interested in automation as well as safety and health-related functions. Also, they watch TV or rest at home except for the housework, so they are interested in TV-related content and environmental setting functions.

“Just rest” is the group with the lowest PCS (44.646) and the lowest health-related quality, so they spend most of their time at home watching TV or relaxing. This group also has high needs for overall smart home functions, where the most needed functions are for health and safety-related functions, while they are also interested in the automation systems and TV content.

“Poor quality of life” is the group with the lowest rate of marriage, the highest rate of living alone, and the lowest basic quality of life. They also have the lowest MCS (45.379) and minimum social events. The necessity for the smart home functions is not high compared to other groups. Among the functions, they need Remote controlled electricity most, and they also require health and safety-related functions.

“Active at home” is the group that spends the most time on a variety of activities at home and has the least time for going out of home. They have the most social events such as visiting outsiders, and the MCS (54.268) is also the highest. They are interested in various smart home functions and devices, and especially the needs of entertainment-related automation/setting are higher than in other groups.

“High quality of life and good health” is centered on men who spend a lot of time out of home. They have the highest PCS (50.733), and they are not very interested in smart home features.

## 4. Discussion

This study conducted a segmentation by RBL to understand the differences in the smart home needs of older people. This contributes to the study on older adults’ lifestyles at home and the study on smart homes with three outcomes. The first is to develop a measurement tool for older people’s RBL. The second is a methodology to build older people segmentation with the RBL tool. Last, we investigated the differences in smart home needs for each segment.

The first contribution is a questionnaire designed to identify the patterns of older adults’ RBL. Since this kind of RBL questionnaire did not exist before, the design took a broad analysis of lifestyle factors for older people from existing research as the starting point [10,12,13,31,32,33,34,35,36]. The proposed RBL questionnaire is composed of seven factors: quality of life, health state-related quality, housework, leisure at home, relaxation at home, need for help in home life, and social event at home. It consists of a total of 25 questions. Kim et al. [46] studied the lifestyle factors of older people when choosing housing, and seven factors were determined: continuous self-improvement, interest in and preference for elder-care facilities, modern family composition, pursuit of trends and hobbies, progressive thinking, and conservative planning. Although it was a lifestyle study targeting older people, it is not really a study of RBL because they had the objective of a housing purchase. In the study for energy saving purposes [40], they designed a tool to measure the housing-related lifestyle. It was based on a total of five factors: acquisition motives, quality aspects, ways of shopping, home improvement, and living situations. Although this is a questionnaire of housing-related lifestyle, it was not tailored to a specific target of old age, and it considered only the energy saving situation. The proposed questionnaire is for older people’s RBL on smart home research. It is differentiated in that it allows us to identify the overall pattern of how older people live at home and to evaluate how satisfied they are with their current life and the areas in which they need help in daily life. The developed questionnaire was translated and shared in English, Korean, and Spanish to be used or further developed for further research (Appendix C: English version).

The second contribution is a methodology to segment into five meaningful and stable groups based on the RBL questionnaire. Segmentation for older people was formed through clustering based on seven RBL factors. And each group was described by its name and characteristics according to the differences in RBL factors: Tired of housework, Just rest, Poor quality of life, Active at home, and High quality of life and good health. Also, it was possible to check the differences in demography among the groups and compare and analyze each group’s health status through PCS and MCS. Looking at similar research, Eissens van der Laan et al. [47] segmented to provide better care for the individual needs of older people and organized five segments: Feeling vital, Difficulties with psychosocial coping, Physical and mobility complaints, Difficulties experienced in multiple domains, and Feeling extremely frail. Since this research is segmented based on health-based needs, it cannot be compared completely, but it can be seen that the mental and physical health states of “High quality of life and good health” based on RBL and “Feeling vital” of the research [47] are similar. Moreover, “Just rest” based on RBL and “Feeling extremely frail” of the research [47] show similar characteristics. This similarity suggests that the health status is strongly influencing residential-based lifestyle. In the study about technology for older people [5], the old age group was segmented into four groups: Healthy and active seniors, People with chronic diseases, People with dementia, and People with mild cognitive impairment. The health state of older adults was also used as the main factor. According to the characteristics of the groups, “healthy and active seniors” of the study [5] and “Active at home” and “High quality of life and good health” based on RBL are considered to be similar. However, since our study was the first to segment old age based on the differences in RBL patterns, it cannot be compared with existing studies exactly. It is also one of the significant results of this study: to develop a new segmentation.

The third contribution is to prove that there are differences in the needs and interests of smart homes for each RBL segment. There are significant differences in 16 out of 26 smart home functions for each group. Also, it was confirmed that the smart home function’s priority and the number of required functions is different. This confirms our hypothesis that the need for a smart home function can be influenced deeply by an individual’s RBL.

## 5. Conclusions

Smart homes can be an important solution to improve the quality of life of individuals while aging. Also, this technology can be promoted at government and public administration levels. Concretely, Seoul Metropolitan Government in South Korea is trying to apply it to older adults [3]. However, for the successful introduction of technology into these adults´ lives, it is necessary to understand their real needs. In this study, clustering of older people was conducted according to RBL, and the differences in smart home function needs for each group were confirmed. It was possible to clearly distinguish those groups that required many functions and those that did not. Namely, in the case of having different RBL, they had different priorities and necessities for smart home functions (Table 6). This study proved that the cluster analysis with RBL provides practical data for smart home research.

We can analyze not only the differences amongst groups but also the similarities. For the groups “Tired of housework”, “Just rest”, and “Active at home”, the needs for smart homes are relatively high, while the needs of “Poor quality of life” and “High quality of life and good health” are relatively low. Although there are slight differences, the functions Call the family in case of emergency, Gas leak detection/smoke detection, Fall detection, and Remote-controlled electricity, are functions required by all groups. The functions Call the family in case of emergency and Fall detection belong to the category “Health and lifestyle monitoring”, while Gas leak detection/smoke detection and Remote-controlled electricity are in the “Intelligent environmental and sustainable services” category (Appendix B). It shows that these two categories and those four functions need to be specially prepared for older users. Although people of the “High quality of life and good health” group are less interested in other functions, they are still interested in health-related functions. Thus, it can be concluded that most of them have concerns about health, including participants who remain healthy. On the contrary, the preference for adding smart functions to existing appliances are not high, such as Smart washing machine, Smart refrigerator, Smart cooker, and Smart coffee maker. This is presumed to be due to the concern for the complexity of the new features compared to that of existing appliances.

In future work, we will explore the causes of these results through qualitative investigation such as interviews, providing a truly in-depth approach to understanding the needs of the elderly, which does not arise with purely quantitative research. Moreover, we will study the relationship analysis of life patterns, health and smart home function needs. And by comparing with other age groups, the characteristics and needs of the older group could be more clearly analyzed and extracted. We also plan to apply this method to a larger number of older people outside South Korea to explore the external validity of our questionnaire and the generalizability of the results.

To the best of the authors’ knowledge, this study is the first attempt to analyze the impact of the elderly’s RBL in the selection of smart home technology. We identified RBL factors for older people and clustered them to compare and analyze the needs of smart home functions, discovering meaningful differences.

The results contained in this paper can contribute to industry and academia. First the industry that develops and sells smart home services can benefit from a deeper knowledge of older users’ needs and the priority of smart home functions in order to provide a more targeted offer. Also, it is possible to find out the characteristics of groups that are interested in smart home functions and those that are not. This allows the target market to be subdivided so that efficient marketing and development can be achieved. Second, this study can also be a good contribution to academia. In the case of research on smart homes for older adults, the study of the segmented older group can be conducted rather than researching one broad mixed group of older people. Identifying this demographic´s detailed needs and life patterns will enable the development of research and products that are more suitable for users. This approach to user analysis will also make the technology more practical.

## Figures and Tables

**Table 1:** Factor analysis of residentially-based lifestyles.

Measurement Items	Quality of Life	House-Work	Need for Help in Home Life	Health State-Related Quality	Leisure at Home	Social Event at Home	Relaxation at Home
Generally, I feel satisfied with my daily life.	0.76						
I am satisfied with how I share my time.	0.68						
I sleep the necessary hours at night.	0.65						
My diet is varied and balanced.	0.54						
I eat at least 3 times a day.	0.46						
Cook or bake		0.76					
Take care of my house		0.66					
Take care of someone		0.54					
Talk to someone					0.33		
I need help to take care of someone			0.7				
I need help to prepare food or buy ingredients.			0.63				
I need help to clean the house, wash the dishes or do the laundry.			0.62				
I need help to wash myself or change my clothes.			0.4				
My chronic disease affects the quality of my daily life.				0.83			
My medication or treatments affect the quality of daily life.				0.79			
I am sleepy during the day.				0.41			
Read books, newspapers or magazines					0.68		
Creative and artistic activities					0.65		
Do light exercise					0.49		
Visits of another type						0.71	
Invite friends						0.71	
Family visit						0.56	
Watch TV or listen to music							0.59
Do nothing because I don’t want to do anything							0.57
Rest							0.57
Cronbach’s alphas	0.7	0.61	0.61	0.69	0.57	0.62	0.49
Eigen value	2.3	2.02	1.9	1.79	1.69	1.64	1.58
Cumulative Var.	0.09	0.17	0.25	0.32	0.39	0.45	0.52

**Table 2:** Results of cluster analysis for residentially-based lifestyle of older people.

Factor	Cluster 1 (*n* = 31)	Cluster 2 (*n* = 57)	Cluster 3 (*n* = 39)	Cluster 4 (*n* = 80)	Cluster 5 (*n* = 64)	*F*-Value
Quality of life	−0.258	0.184	−1.575	0.282	0.568	24.44 ***
House work	0.848	−0.334	−0.188	0.437	−0.546	10.52 **
Need for help in home life	1.755	−0.187	−0.557	−0.378	0.128	31.88 ***
Health state−related quality	−0.131	−0.925	0.100	0.062	0.749	111.4 ***
Leisure at home	−0.405	−0.374	−0.235	0.930	−0.490	6.155 *
Social event at home	−0.019	0.135	−0.434	0.338	−0.269	0.661
Relaxation at home	−0.054	0.888	−0.364	−0.140	−0.368	54.31 ***

*** *p* < 0.001, ** 0.001 ≤ *p* < 0.01, * 0.01 ≤ *p* < 0.05.

**Table 3:** Demographic characteristics of each cluster.

Demographic Characteristics		Cluster 1 *n* = 31 (11.4%)	Cluster 2 *n* = 57 (21.0%)	Cluster 3 *n* = 39 (14.4%)	Cluster 4 *n* = 80 (29.5%)	Cluster 5 *n* = 64 (23.6%)	χ^2^
		Tired of Housework	Just Rest	Poor Quality of Life	Active at Home	High Quality of Life and Good Health	
Gender	Male	11 (33.5)	27 (47.4)	15 (38.5)	25 (31.3)	38 (59.4)	13.005 *
	Female	20 (64.5)	30 (52.6)	24 (61.5)	55 (68.7)	26 (40.6)	
Age	65–69	16 (51.6)	23 (40.4)	23 (59)	40 (50)	36 (56.3)	9.3738
	70–74	8 (25.8)	23 (40.4)	14 (35.9)	25 (31.3)	18 (28.1)	
	75–79	6 (19.4)	9 (15.8)	1 (2.6)	13 (16.3)	9 (14.1)	
	>80	1 (3.2)	2 (3.6)	1 (2.6)	2 (2.4)	1 (1.5)	
Marital status	Married	28 (90.3)	51 (89.5)	30 (76.9)	69 (86.3)	60 (93.8)	18.16
	Single	0 (0)	0 (0)	1 (2.6)	0 (0)	0 (0)	
	Widowed	3 (9.7)	6 (10.5)	6 (15.4)	11 (13.8)	3 (4.7)	
	Separated	0 (0)	0 (0)	2 (5.1)	0 (0)	1 (1.6)	
Resident type	Alone	1 (3.2)	4 (7)	6 (15.4)	5 (6.3)	2 (3.1)	14.122
	With Spouse	27 (87.1)	43 (75.4)	23 (59)	59 (73.8)	53 (82.8)	
	With Family	3 (9.7)	10 (17.5)	10 (25.6)	15 (18.8)	8 (12.5)	
	Others	0 (0)	0 (0)	0 (0)	1 (1.3)	1 (1.6)	
Caregiver	Spouse	26 (83.9)	48 (84.2)	29 (74.4)	66 (82.5)	58 (90.6)	18.954
	Sons or daughters	3 (9.7)	5 (8.8)	9 (23.1)	12 (15)	4 (6.3)	
	Other family	2 (6.5)	3 (5.3)	0 (0)	2 (2.5)	1 (1.6)	
	Friends	0 (0)	1 (1.8)	0 (0)	0 (0)	0 (0)	
	Others	0 (0)	0 (0)	1 (2.6)	0 (0)	1 (1.6)	
Spent time out of home	less than 1	2 (6.5)	7 (12.3)	5 (12.8)	8 (10)	4 (6.3)	19.487
	1–3	19 (61.3)	32 (56.1)	14 (35.9)	39 (48.8)	23 (35.9)	
	3–6	4 (12.9)	8 (14)	11 (28.2)	24 (30)	18 (28.1)	
	more than 6	6 (19.4)	10 (17.5)	9 (23.1)	9 (11.3)	19 (29.7)	
Education	Basic	3 (9.7)	2 (3.5)	3 (7.7)	1 (1.3)	2 (3.1)	10.304
	Intermediate	13 (41.9)	20 (35.1)	19 (48.7)	28 (35)	21 (32.8)	
	University	15 (48.4)	35 (61.4)	17 (43.6)	51 (63.8)	41 (64.1)	
Income (million won)	less than 1	0 (0)	6 (10.5)	3 (7.7)	5 (6.3)	0 (0)	28.126 *
	1–2	8 (25.8)	5 (8.8)	11 (28.2)	12 (15)	8 (12.5)	
	2–3	12 (38.7)	13 (22.8)	13 (33.3)	15 (18.8)	20 (31.3)	
	more than 3	11 (35.5)	33 (57.9)	12 (30.8)	48 (60)	36 (56.3)	

* 0.01 ≤ *p* < 0.05.

**Table 4:** Cluster differences by health score.

Health (SF 12v2)	Cluster 1	Cluster 2	Cluster 3	Cluster 4	Cluster 5	*F*-Value
	Tired of Housework	Just Rest	Poor Quality of Life	Active at Home	High Quality of Life and Good Health	
Physical component summary	44.915	44.646	46.273	48.868	50.733	24.48 ***
Mental component summary	48.272	50.914	45.379	54.268	53.704	15.75 ***

*** *p* < 0.001.

**Table 5:** The needs for smart home functions for each cluster.

Smart Home Functions	Cluster 1	Cluster 2	Cluster 3	Cluster 4	Cluster 5	*F*-Value
	Tired of Housework	Just Rest	Poor Quality of Life	Active at Home	High Quality of Life and Good Health	
**Smart washing machine**	2.097	2.123	2.103	1.938	1.766	3.824
Smart refrigerator	2.387	2.246	2.077	2.313	1.906	2.663
Smart cooker and smart coffee maker	2.355	2.368	1.821	2.450	1.859	2.65
Schedule reminder	2.839	2.842	2.821	2.863	2.422	2.849
Reminder to take medicine	2.871	2.842	2.487	2.563	2.000	13.96 ***
Open windows/doors detection	2.387	2.509	2.359	2.575	2.031	1.893
Automatic watering in the garden	2.323	2.175	1.846	2.175	1.984	1.103
Automatically checked postbox	2.645	2.596	2.282	2.463	2.109	6.27 *
Automatic heating	3.000	3.070	2.564	2.738	2.188	14.58 ***
Gas leak detection/smoke detection	3.871	3.368	3.077	3.388	2.719	10.87 **
Remote controlled electricity	3.516	3.298	3.256	3.400	2.625	9.04 **
Remote controlled lights	3.194	3.000	2.769	2.913	2.422	8.766 **
Light mode settings	2.774	2.772	2.410	2.863	2.188	4.552 *
TV auto-play function, notifications from my channel	2.710	2.526	2.410	2.638	2.109	4.163 *
Personalized learning TV content	2.710	2.737	2.641	2.975	2.469	0.265
Automatic cinema mode setting	2.613	2.684	2.256	2.813	2.109	3.255
Fall detection	3.548	2.982	2.974	3.188	2.641	5.138 *
Monitoring in your absence	2.645	2.526	2.385	2.638	2.281	0.985
Measure sleep health	3.065	2.877	2.846	2.988	2.469	3.476
Sensors you can wear as clothes	2.871	2.842	2.513	2.800	2.313	4.077 *
Mental health detection	3.355	2.982	2.923	3.050	2.422	8.981 **
Telehealthcare	3.419	3.316	2.821	3.238	2.484	11.01 **
Home Management Assistant Robot	2.710	3.070	2.308	2.975	2.219	4.742 *
Equipment to help you up and down stairs	2.645	2.632	2.308	2.375	1.969	10.46 **
Robot like a friend	2.323	2.281	1.718	1.863	1.719	11.5 ***
Call the family in case of emergency	3.581	3.351	3.128	3.225	2.844	6.67 *

*** *p* < 0.001, ** 0.001 ≤ *p* < 0.01, * 0.01 ≤ *p* <0.05.

**Table 6:** The priority of smart home functions for each cluster.

Priority	Cluster 1	Cluster 2	Cluster 3	Cluster 4	Cluster 5
	Tired of Housework	Just Rest	Poor Quality of Life	Active at Home	High Quality of Life and Good Health
1	Gas leak detection/smoke detection	Gas leak detection/smoke detection	Remote controlled electricity	Remote controlled electricity	Call the family in case of emergency.
2	Call the family in case of emergency.	Call the family in case of emergency.	Call the family in case of emergency.	Gas leak detection/smoke detection	Gas leak detection/smoke detection
3	Fall detection	Telehealthcare	Gas leak detection/smoke detection	Telehealthcare	Fall detection
4	Remote controlled electricity	Remote controlled electricity	Fall detection	Call the family in case of emergency.	Remote controlled electricity
5	Telehealthcare	Automatic heating	Mental health detection	Fall detection	
		Home Management Assistant Robot			
6	Mental health detection	Remote controlled lights	Measure sleep health	Mental health detection	
7	Remote controlled lights	Fall detection	Schedule reminder	Measure sleep health	
		Mental health detection	Telehealthcare		
8	Measure sleep health	Measure sleep health	Remote controlled lights	Personalized learning TV content	
				Home Management Assistant Robot	
9	Automatic heating	Schedule reminder	Personalized learning TV content	Remote controlled lights	
		Reminder to take medicine			
		Sensors you can wear as clothes			
10	Schedule reminder	Light mode settings	Automatic heating	Schedule reminder	
				Light mode settings	
11	Reminder to take medicine	Personalized learning TV content	Sensors you can wear as clothes	Automatic cinema mode setting	
	Sensors you can wear as clothes				
12	Light mode settings	Automatic cinema mode setting		Sensors you can wear as clothes	
13	TV auto-play function, notifications from my channel	Equipment to help you up and down stairs		Automatic heating	
	Personalized learning TV content				
	Home Management Assistant Robot				
14	Automatically checked postbox	Automatically checked postbox		TV auto-play function, notifications from my channel	
	Monitoring in your absence			Monitoring in your absence	
	Equipment to help you up and down stairs				
15	Automatic cinema mode setting	TV auto-play function, notifications from my channel		Open windows/doors detection	
		Monitoring in your absence			
16		Open windows/doors detection		Reminder to take medicine	

## Appendix A

**Table A1:** Lifestyles of older people.

Paper	Lifestyle Indices for Older People
Bone [10]	Discretionary Income/Health/Activity level/Discretionary time/Response to other people
Moschis et al. [12]	Biophysical/Psychological/Social circumstances/Key life events
Sudbury and Simcock [13]	Health and activities/Social measures/Psychological measures/Consumer behaviors, attitudes and values/Media usage
Du [31]	Physical health (Physical condition, Eating routine, Exercise habits, Quality of sleep, Use of drugs or alcohol, Living environment/Mental health (Mental status, Emotion control, Stress handling, Self-regulation)/Social wellbeing (Prosocial behaviors, Interpersonal relationships, Social recognition)
Yuan et al. [35]	Substance abuse (Cigarette, Alcohol)/Leisure activity (TV watching, Reading, Smartphone use, Social activity, Exercise)
Shahboulaghi et al. [34]	Interpersonal and social relationships/Stress management/Eating/Exercise/Prevention
Zhang et al. [36]	Smoking/Drinking/Diet status/Sleep status/Exercise & labor status/Leisure time activities
Saint Martin et al. [33]	Smoking/Alcohol Consumption/Health Care/Physical Activity/Intellectual Activity/Social Activity
Fallah Mehrabadi et al. [32]	Prevention/Physical Activity/Recreation and Entertainment/Healthy Nutrition/Stress Management/Social and Interpersonal Relationships

## Appendix B

**Table A2:** Smart home functions.

Service	Category	Function	Description/Scenario
**Comfort**	Automation of daily routines	Smart washing machine	You can program it to finish anytime you want, and you can see the progress on your phone.
		Smart refrigerator	Through the refrigerator screen, you can see the inside of the refrigerator, and manage the expiration date of each food item. The display also allows you to buy food ingredients directly online.
		Smart cooker and smart coffee maker	Cooking is done at the desired time and is kept warm in storage mode until you consume it. It also makes coffee automatically at the time that you want.
		Schedule reminder	It tells you not to forget the main events of the day.
		Reminder to take medicine	It checks the time and amount of medicine that you usually take and reminds you to take it.
	Remote home management	Opened window/door detection	You can check if the windows and the door are open or closed on your phone. And you can close it on your phone.
		Automatic watering in the garden	You can water your garden anytime with the remote control. During traveling, you can check the state of the garden on your mobile screen.
		Automatically checked postbox	It will notify you when a new letter arrives. And you can see by mobile how much correspondence is in the mailbox.
	Intelligent environmental and sustainability services	Automatic heating	If you are at home, the heating turns on automatically according to the set temperature. It can also be controlled with remote control.
		Gas leak detection/smoke detection	It detects and alerts you if there is smoke or gas leak. And if you are not at home, you will receive a notification on your phone.
		Remote controlled electricity	The use of electricity can be reduced by cutting off power before going to bed or going out. You don’t have to unplug appliances every time you leave.
		Remote controlled lights	You can turn the lights on or off in several spaces at once. You can also remotely control the lights in the room you want.
		Light mode settings	The brightness of the lamp is set to a preset mode, such as TV viewing mode, reading mode, eating mode, etc.
	Smart leisure	TV auto-play function, notifications from my channel	You can receive program information from the channels you watch frequently. If you set the program you want, the TV automatically plays the program at that time.
		Personalized learning TV content	You can watch the content you want to learn on television.
		Automatic cinema mode setting	You can watch TV shows or movies with your favorite screen brightness and volume. You can also set the brightness of external lights or blinds at the same time.
Monitoring	Health and lifestyle monitoring	Fall detection	If you fall, your family or your caregiver will receive a notification.
		Monitoring in your absence	There is a camera that allows you to see inside your home, and it checks on the status of your pet while you are away.
		Measure sleep health	When you sleep in the usual bed, your heart rate and sleep quality will be measured automatically.
		Sensors you can wear as clothes	It is a light cloth. If you wear it daily, you can verify your health status in real-time and communicate with the hospital according to the data.
		Mental health detection	It checks your usual mental state and notifies you if something is wrong.
		Call the family in case of emergency	Automatically calls family and friends in case of emergency.
Health therapy	Remote interaction	Telehealthcare	You can talk to your doctor and get medical consultation on video from home without having to go to the hospital.
Support	Assist mobility	Home management assistant robot	It helps with physically demanding tasks such as cleaning and transporting.
		Equipment to help up and down stairs	Reduce physical tension when going up and down stairs or when walking.
	Support with socialization	Robot like a friend	You can talk or play card games with the robot as if it was your friend.

## Appendix C

**Table A3:** Residentially-based lifestyle questionnaire.

Frequency	Rarely	Sometimes	Often	Almost Always	Always
I sleep the necessary hours at night.					
I am sleepy during the day.					
I eat at least 3 times a day.					
My diet is varied and balanced.					
My chronic disease affects the quality of my daily life.					
My medication or treatments affect the quality of my daily life.					
Generally, I feel satisfied with my daily life.					
I need help to clean the house, wash the dishes or do the laundry.					
I need help to wash myself or change my clothes.					
I need help to prepare food or buy ingredients.					
I need help to take care of someone (grandchild, child, brother, spouse).					
**Time spent**	**Never**	**Less Than 1 h Daily**	**About 1 h Daily**	**About 2 h Daily**	**More than 3 h Daily**
Watch TV or listen to music					
Take care of someone (grandchild, child, brother, spouse)					
Take care of my house (clean, care for the garden or plants)					
Read books, newspapers or magazines					
Cook or bake					
Do light exercise					
Talk to someone (including call)					
Rest					
Do nothing because I don’t want to do anything					
Creative and artistic activities (sew, paint, decorate, write...)					
**Frequency**	**Less Than 1 Time Per 1 month**	**1 Time Per 1 Month**	**2 Times Per 1 Month**	**3 Times Per 1 Month**	**More Than 4 Times Per 1 Month**
Invite friends					
Family visit					
Visits of another type					
**S** **atisfaction**	**Not at All**	**A Little Bit**	**Moderately**	**Quite A Bit**	**Extremely**
I am satisfied with how I share my time.					
